# Colonic perianastomotic carcinogenesis in an experimental model

**DOI:** 10.1186/1471-2407-8-217

**Published:** 2008-07-31

**Authors:** Sergio Pérez-Holanda, Luis Rodrigo, Carme Pinyol-Felis, Joan Vinyas-Salas

**Affiliations:** 1 Department of Surgery, Hospital Valle Del Nalón, Langreo, Asturias, Spain; 2 Gastroenterology Service, School of Medicine, University of Oviedo, Asturias, Spain; 3 Departments of Internal Medicine and Surgery, School of Medicine, Hospital "Arnau de Vilanova", University of Lleida, Catalonia, Spain

## Abstract

**Background:**

To examine the effect of anastomosis on experimental carcinogenesis in the colon of rats.

**Methods:**

Forty-three 10-week-old male and female Sprague-Dawley rats were operated on by performing an end-to-side ileorectostomy. Group A:16 rats received no treatment. Group B: 27 rats received 18 subcutaneous injections weekly at a dose of 21 mg/kg wt of 1–2 dimethylhydrazine (DMH), from the eighth day after the intervention. Animals were sacrificed between 25–27 weeks. The number of tumours, their localization, size and microscopic characteristics were recorded. A paired chi-squared analysis was performed comparing tumoral induction in the perianastomotic zone with the rest of colon with faeces.

**Results:**

No tumours appeared in the dimethylhydrazine-free group. The percentage tumoral area was greater in the perianastomotic zone compared to tumours which had developed in the rest of colon with faeces (p = 0.014).

**Conclusion:**

We found a cocarcinogenic effect due to the creation of an anastomosis, when using an experimental model of colonic carcinogenesis induced by DMH in rats.

## Background

Colorectal cancer (CRC) is a frequent tumour, with an elevated mortality in western countries [1]. It is the second cancer in the population of Asturias (Northern Spain), and represents 13% of the total number of cases of cancer diagnosed annually. CRC causes 3.7% of the total number of deaths and 14.1% of the deaths due to cancer [2].

To study the influence of the different etiological environmental factors on colon cancer in man, long-term prospective studies are difficult to carry out [3].

Several authors have suggested that colon cancer is promoted by non-specific colonic lesions [4,5]. Late anastomotic recurrences of colorectal carcinoma could be explained by this hypothesis [6-9].

The induction of colorectal cancer with 1–2 dimethylhydrazine (DMH) in rats is a currently valid experimental model, which is transferable to humans, both in their macro-microscopic and biological behaviour [10-12].

The aim of the present study was to investigate the effect of anastomosis in the colon of rats subjected to colonic tumoral induction with DMH.

## Methods

This study is part of a previously published experiment in which end-to-side ileorectal anastomosis, constructed with 4-0 silk suture, was performed in rats, as described elsewhere [13].

The intervention time was approximately 35 minutes and rats were returned to their cages, and placed in a lateral position.

The commencement of oral tolerance was permitted from the time of wakening. The reintroduction of dietary pellets in the dispensers was made 12 hours later. Faeces of soft consistency and normal colour appeared at 48 hours. DMH injections were started on day eight after the intervention [6,14].

Both male and female rats were used in this study. This being based on the differences found between them in previous studies [13], where female rats showed a lower overall mortality rate, and a lower incidence of colorectal cancer. A higher number were tumour-free and a trend toward a smaller size in tumours developed in defunctionalized colonic segments was seen. Their behaviour seemed to be different from that in male animals.

Sixty-eight 10-weeks-old male and female Sprague-Dawley rats of identical strain from our laboratory were operated on. Twelve rats were dead within 30 postoperative days, and another 13 rats died before completion of the study. Thus 43 rats out of 68 were studied.

The 43 surviving rats that finished the study were distributed into two groups: Group A: 16 rats received no treatment. Group B: 27 rats, both male and female, received 18 subcutaneous injections weekly at a dose of 21 mg/kg. wt. of 1–2 dimethylhydrazine (DMH; Fluka Chemica A.G., Sigma Co.^®^, St. Louis, Missouri, USA). The DMH solution was prepared weekly as previously reported [13,15,16]. Injections were administered into the lumbar region from the eighth day after intervention.

The diet of rats (ITM-R20 diet, Lab Letica ^®^, Barcelona, Spain) contained 3 % fat and 5 % fibre. The quantity of food consumed was controlled throughout the study. Fifty percent of the animals from each group were weighed weekly, until sacrificed. Room conditions were maintained at a constant temperature and humidity, and a circadian cycle of light-darkness of 12 hours was established [17,18]. The animals were separated in cages with a maximum of three per cage, in order to avoid autophagia. Animals of different gender were not mixed in order to avoid aggressions, similar to those found in previous studies [13,15,16,19,20].

Both the recommendations of the European Ethics Committee (E.C. Directive 1986/609) and the Spanish Royal Decree 1201/2005, October 10 (BOE number 25, October 21, 2005) for animal handling in experimental conditions were followed throughout the study.

Survival rats were sacrificed between 25–27 weeks of study, by means of intra-peritoneal injection of a lethal dose of 4.5% chloral hydrate. A fixed and equal number of rats from each group were sacrificed each week [15,16].

At necropsy, the thoracic and abdominal cavities were examined. The number of tumours, their localization and size were recorded. Samples of tumours were later taken for histological study, both from left colon with faeces and rectum, and from the perianastomotic zone. These samples were fixed and prepared by means of hematoxyline-eosine staining. The lesions of the colonic mucosa were classified according to the criterion of Grau Castro-Piqué Badía [21] and Lev [22]. Other findings were also recorded.

The length of the colonic segments was measured [23] in order to obtain the colonic segment surface. Tumour size was obtained by measuring maximum diameters, and considered as the mean ± standard deviation (SD) [13,16,24]. The perianastomotic zone was defined as 2 cm above and below the anastomotic line [6,7]. The global tumoral area is presented in square centimetres. The percentage tumoral area is defined as the relationship between each tumoral area and its colonic segment surface (per cent).

We have previously demonstrated [13] that the absence of faeces could modify the carcinogenic effect of DMH. Thus tumours which developed in the defunctionalized colonic segments were excluded from the study.

Data were analysed using the SPSS package, performing analysis of variance (ANOVA) and a paired chi-squared test. Differences were considered as significant when the p value was less than or equal to 0.05.

## Results

Twenty-five rats (36.8%) died prior to completion of the study, 10 rats (38.5%) from group A and 15 (35.7%) from group B. Similar percentages of deaths have been described in previous studies [13].

The postoperative mortality (within 30 days) of the rats operated on was 17.6% (12 out of 68).

No tumours were observed in rats from group A. Morphological description of the sample, and tumour incidence and distribution are shown in Table 1.

**Table 1:** Morphological description of sample, tumour incidence and distribution.

	SP	SP + DMH
Weight in g (mean ± SD)		
Males	600 ± 0.00	521.67 ± 108.89
Females	291 ± 42.22	314.44 ± 49.67

Number of rats sacrificed, n (%)	16 (61.5 %)	27 (64.3 %)
Males/Females	1/15	9/18

Number of tumour-free rats, n (%)	16 (100 %)	9 (33.3 %)
Males, n (%)	1/1 (100 %)	0/9 (0%)
Females, n (%)	15/15 (100 %)	9/18 (50 %) *

Total number of tumours, n	-	29
Males/Females, n	-	15/14

Mean number of tumours/rat (mean ± SD)		
Males	-	2.22 ± 0.83
Females	-	2.11 ± 1.17

Tumour area in cm2 (mean ± SD)		
Males	-	7.38 ± 0.97
Females	-	6.85 ± 0.90

Males *vs*. Females: * within SP+DMH group, p = 0,011.

DMH: Dimethylhydrazine; g: grams; n: number; SD: Standard deviation; SP: Surgical procedure.

Twenty-nine tumours were studied in the 27 rats (Group B) that finished the study. In these rats, 34.5% of tumours were located in the perianastomotic zone (10 out of 29), while 65.5% were located in the left colonic segments with presence of faeces (19 out of 29) (p = 0.90).

Taking all the animals together, the global tumoral area was 22.69 cm^2^. The tumoral area in the perianastomotic zone was 13.37 cm^2 ^(58.23% of the whole tumoral area; X = 133.67 ± 141.31 mm^2^), and in the "extra-anastomotic" zone (the rest of the colonic segments with faeces) was 9.32 cm^2 ^(41.77%; X = 49.06 ± 92.19 mm^2^) (p = 0.13). No significant differences were found between male and female rats when comparing the mean number of tumours per rat, excluding tumour-free rats. Moreover, no differences were observed in the tumoral area developed in male rats compared to female rats in group B (Table 1).

In the animals that completed the study, the global colonic tissue examined was 113.75 cm^2^, and was distributed as follows: 19.58 cm^2 ^(17.24% of the whole tissue surface) of perianastomotic tissue, 60.26 cm^2 ^(52.95%) of defunctionalized descending colon with faeces and 33.91 cm^2 ^(29.81%) of rectum surface. The extra-anastomotic tissue surface represented 94.17 cm^2 ^(82.76%).

With respect to percentage tumoral area, 22.69 cm^2 ^out of 113.76 cm^2 ^of colonic tissue had changed to tumoral tissue (19.94%). This transformation was 68.25% in the perianastomotic zone and 9.90% in the rest of the large bowel with faeces (p = 0.014) (Table 2).

**Table 2:** Differences between tumours located in the perianastomotic zone and tumours located in the rest of colon with faeces in rats of group B.

	Perianastomotic zone	Colon with faeces
Number of tumours, n	10	19
Average tumour size, mm^2^	133.67 ± 141.31	49.06 ± 92.19
Tumoral area, cm^2^	13.37	9.32
Percentage tumoral area, %	68.25 *	9.90

*Significant differences observed, p = 0.014.

In relation to the microscopic characteristic of the tumours, 5 mucinous carcinomas (2 in perianastomotic zone and 3 in the extra-anastomotic colic area) and 24 adenocarcinomas were observed. One male rat developed peritoneal carcinomatosis, and a female rat showed multiple liver and spleen metastases which were microscopically confirmed.

## Discussion

The overall mortality rate for experimental studies in rats, which includes surgical procedures, is high. For this reason, their performance is low [25-27]. In our study we lost a total of 36.8% of the animals, despite the fact that our postoperative mortality rate was low and appropriate [28-30].

Some authors have reported an incidence of spontaneous experimental colonic carcinogenesis of less than 2–3 rats/100.000 rats observed [31]. Thus we expected to find tumour-free animals in the absence of carcinogen (DMH). Based on the number of animals that completed the study, the number of rats in each group was statistically designed to obtain the minimum number of rats in the group in which no tumours were expected (group A). A paired statistical analysis was then performed in those rats that developed colonic tumours and a comparison of such was made according to their location.

The model of colonic carcinogenesis with 1,2-dimethylhydrazine (DMH) in rats is a valid model, and is transferable to humans [10-12]. DMH has mutagenic properties [32] with an additive effect both through blood and luminal route [10].

We have previously demonstrated [13] that the absence of faeces could modify the carcinogenic effect of DMH. Thus, those rats in which tumours developed in the defunctionalized colonic segments (in absence of faeces) were excluded from the study.

The perianastomotic zone was defined as being 2 centimetres above and below the anastomotic line, as suggested by Noguera et al. [6]. We had previously used [19,23] a different definition of perianastomotic zone, which was defined as being 5 mm above and below the anastomotic line. The greater surface of colonic tissue used in this study, makes results implement their adequacy [6,7,23].

Taking tumour size as a parameter to evaluate the carcinogenic effect of an anastomosis from tumours in the perianastomotic zone compared to tumours located in the rest of colon with faeces, no significant differences were observed between the mean values, despite a big difference between them. Thus tumour size was a less reliable parameter than others when evaluating the mucosal carcinogenic changes because of its wide variability related to both colon size and tumoral tissue. A lower number of tumours were found in the perianastomotic zone compared to the rest of colon with faeces in rats of group B.

Some studies have recommended both percentage and global tumoral area as more adequate parameters than the number of tumours to evaluate tumoral colonic proliferation [7]. Based on this recommendation, we found that our rats showed greater tumoral areas (with no significant differences), and their percentage tumoral areas were significantly greater in the perianastomotic zone compared to the rest of the colon with faeces in rats from group B (p = 0.014).

As in previous studies [13], we observed different tumoral behaviour in relation to gender. In fact, female rats showed a lower overall mortality rate, a lower incidence of colorectal cancer, and a higher number were tumour-free. Nevertheless, no differences were observed in tumoral induction between male and female rats. This being defined in terms of the total number of tumours, the mean number of tumours per rat, and the tumoral area.

Thus, a cocarcinogenic effect due to anastomosis might have been observed in the colon of those rats subjected to colonic tumoral induction with DMH, and gender did not modify this effect.

Late anastomotic recurrences of colorectal carcinoma are an important risk in patients who undergo procedures that include colorectal anastomosis. Resulting from an increased cell proliferation at colonic crypts bordering the anastomotic line [4], an increased cellular activity near the anastomosis [23], and from a chronic inflammatory effect by suture [5-9,33], the hypothesis of a higher susceptibility of the anastomotic mucosa represents the most favourable explanation for these recurrences.

This chronic inflammatory effect by suture may be modulated depending on the materials used [19]. In all rats, anastomosis were constructed with a 4-0 silk suture, which could be confirmed on autopsy. The non-absorbable material used might have induced a continuous and higher inflammatory effect, based on an elevated crypt cell production by silk [34], compared to other suture materials [5]. Thus, the use of silk sutures might have increased the cocarcinogenic effect of anastomosis in our animals.

## Conclusion

Based in our experimental findings, we can conclude that a cocarcinogenic effect of anastomosis constructed with silk suture has been observed in the colon with faeces of both male and female Sprague-Dawley rats, when using an experimental model of colonic carcinogenesis (18 subcutaneous injections weekly at a dose of 21 mg/kg. wt. of 1–2 dimethylhydrazine).

## Competing interests

The authors declare that they have no competing interests.

## Authors' contributions

Prof. Luis Rodrigo, MD, PhD, has contributed in the conception and revision of the discussion, selection and approval of references used, and supervision-approval of the final manuscript. Dr. Carme Pinyol-Felis, PhD, has contributed into the surgical performance phase, so as in acquisition of data, bibliography collection, and macro-microscopic study of samples. Prof. Joan Vinyas-Salas; MD, PhD, has designed the study, contributed in revision of the results, and supervised the surgical procedure technique. At last, Dr. Sergio Perez-Holanda, MD, PhD, has contributed in the conception of the study, so as in the collection, analysis and interpretation of data, team's coordination, and has written and draft the manuscript.

All authors have read and approved the final manuscript.

## Pre-publication history

The pre-publication history for this paper can be accessed here:



## References

[B1] Greenwald P (1992). Colon cancer overview. Cancer.

[B2] Rayón Suárez C (2005). Programa de Atención al Cáncer 2005–2007.

[B3] Weisburger JH (1991). Causes, relevant mechanisms and prevention of large bowel cancer. Semin Oncol.

[B4] Pozharisshi KM (1975). The significance of non specific injury for colon carcinogenesis in rats. Cancer Res.

[B5] Rokitansky A, Trubel W, Buxbaum P, Moeschl P (1989). 1,2 Dimethylhydrazine-induced carcinogenesis influenced by different colonic anastomoses in rats. Eur Surg Res.

[B6] Noguera Aguilar JF, Tortajada Collado C, Moron Canis JM, Plaza Martínez A, Amengual Antich I, Pujol Tugores JJ (2002). Modelo experimental para el estudio de la recidiva locorregional del cáncer colorrectal. Rev Esp Enferm Dig.

[B7] Noguera Aguilar JF, Amengual Antich I, Plaza Martínez A, Tortajada Collado C, Morón Canis JM, Pujol Tugores JJ (2004). Influencia de la manipulación quirúrgica del colon en la carcinogénesis cólica inducida en ratas. Rev Esp Enferm Dig.

[B8] Appleton GVN, Davies PW, Williamson RCN (1990). El efecto de la desfuncionalización sobre la citocinética y el cáncer en las líneas de sutura del colon. Br J Surg.

[B9] Rubio CA, Nylander G (1982). Surgical resection of the rat colon: effects on carcinogenesis by 1,2-Dimethylhydrazine. J Natl Cancer Inst.

[B10] Banerjee A, Quirke P, Path FRC (1998). Experimental models of colorectal cancer. Dis Colon Rectum.

[B11] Barkla DH, Tutton PJ (1978). Ultrastructure of 1,2-dimethylhydrazine-induced adenocarcinomas in rat colon. J Natl Cancer Inst.

[B12] Druckrey H, Preussman R, Matzkies F, Barkla DH, Tutton PJ (1978). Selektive Erzeugung von Darmkaebs bei Ratten durch 1, 2-Dimethylhydrazin. Ultrastructure of 1, 2-dimethylhydrazine-induced adenocarcinomas in rat colon J Natl Cancer Inst.

[B13] Perez-Holanda S, Rodrigo L, Viñas-Salas J, Piñol-Felis C, Ildefonso C (2006). Effect of defunctionalization on colon carcinogenesis in the rat. Rev Esp Enferm Dig.

[B14] Chang WW (1982). Morphological basis of multistep process in experimental colonic carcinogenesis. Virchows Arch B Cell Pathol Incl Mol Pathol.

[B15] Viñas Salas J, Biendicho Palau P, Piñol Felis C, Miguelsanz García S, Perez-Holanda S (1998). Calcium inhibits colon carcinogenesis in an experimental model in the rat. Eur J Cancer.

[B16] Pérez-Holanda S, Rodrigo L, Viñas-Salas J, Piñol-Felis C (2005). Effect of ethanol consumption on colon cancer in an experimental model. Rev Esp Enferm Dig.

[B17] Hardman WE, Cameron IL (1994). Colonic crypts located over lymphoid nodules of 1,2-dimethylhydrazine-treated rats are hyperplastic and at high risk of forming adenocarcinomas. Carcinogenesis.

[B18] Glauert HP, Bennink MR (1983). Influence of diet or intrarectal bile acid injections on colon epithelial cell proliferation in rats previously injected with 1,2-dimethylhydrazine. J Nutr.

[B19] Buenestado García J, Reñé Espinet JM, Piñol Felis C, Viñas Salas J (2002). Modelo experimental de cáncer colorrectal. Rev Esp Enferm Dig.

[B20] Buenestado García J, Piñol Felis C, Reñé Espinet JM, Pérez-Holanda S, Viñas Salas J (2002). Silk suture promotes colon cancer in an experimental carcinogenic model. Dig Liver Dis.

[B21] Grau de Castro JJ, Piqué Badía JM, (Eds) (1990). Cáncer colorrectal. Monografías clínicas en oncología.

[B22] Lev R (1990). Adenomatous polyps of the colon: Pathological and clinical features.

[B23] Viñas-Salas J, Piñol Felis C, Fermiñán A, Egido R, Pérez-Holanda S, Biendicho P, Buenestado J, Reñé Espinet JM (2001). Repetitive mucosal trauma promotes colon cancer in experimental rat model of colonic carcinogenesis. Rev Esp Enferm Dig.

[B24] Ma Q, Hoper M, Anderson N, Rowlands BJ (1996). Effect of supplemental L-arginine in a chemical-induced model of colorectal cancer. World J Surg.

[B25] Zhang J, Lam LK (1994). Colonoscopic colostomy model in rats for colon tumorigenesis studies. Carcinogenesis.

[B26] Morvay K, Szentléleki K, Török G, Pintér A, Börzsönyi M, Nawroth R (1989). Effect of change of faecal bile acid excretion achieved by operative procedures on 1,2-dimethylhydrazine-induced colon cancer in rats. Dis Colon Rectum.

[B27] Tempero MA, Zetterman RK (1987). Effects of sodium cholate on experimental carcinogenesis and cell proliferation in an excluded colonic segment. J Surg Oncol.

[B28] Celik C, Mittelman A, Paolini NS, Lewis D, Evans JT (1981). Effects of 1,2-symmetrical-dimethylhydrazine on jejunocolic transposition in Sprague-Dawley rats. Cancer Res.

[B29] Heppell J, deZubiria M, Brais MF, Durh MA, Carioto S, Boivin Y, Potvin C (1990). An assessment of the risk of neoplasia in long-term ileal resevoirs using the DMH-rodent model. Dis Colon Rectum.

[B30] Bristol JB, Davies PW, Williamson RCN, Malt RA, Williamson RCN (1982). Subtotal jejunoileal bypass enhances experimental colorectal carcinogenesis unless weight reduction is profound. Colonic carcinogenesis.

[B31] Winkler R, Pfeiffer M, Ayisi K, A Dörner A, Malt RA, Williamson RCN (1982). Spontaneous colostomy cancer in rat: a handily model of colonic carcinogenesis. Colonic carcinogenesis.

[B32] Wakabayashi K, Nagao M, Esumi H, Sugimura T (1992). Food-derived mutagens and carcinogens. Cancer Res.

[B33] Noguera Aguilar JF, Zurita Romero M, Tortajada Collado C, Amengual Antich I, Soro Gosalvez JA, Rial Planas R (2000). Perianastomotic colonic tumors after inclusion of titanium or Lactomer in the anastomotic suture line. An experimental study in rats. Rev Esp Enferm Dig.

[B34] McCue JL, Phillips RK (1993). Cellular proliferation at sutured and sutureless colonic anastomoses. Dis Colon Rectum.

